# Odonto-Chirurgical Society of Scotland

**Published:** 1887-06

**Authors:** 


ARTICLE II.

### ODONTO-CHIRURGICAL SOCIETY OF SCOTLAND.

The Annual General Meeting of the Society was held in Edinburgh, on March 11th, the President (Mr. W. Bowman Macleod, L. D. S. Edin.) in the chair.

The Treasurer (Mr. M. Macgregor) submitted his report, which showed an income of £49 9s. 10d., derived from subscriptions, entry money, and interest on bank account. This, with a balance carried forward from the previous year, and a deposit receipt of £95, gave a total of £146 10s. 10½d. After deduction of expenses, which included printing, rent of rooms, stationery, &c., a sum of £112 7s. 0½d. was left as representing the funds of the Society—an increase of £15 6s. over the corresponding amount of the previous year.

The Curator and Librarian (Mr. G. W. Watson), in tendering his report, said he had great pleasure in announcing that Mr. Robert Hepburn, of London, had very generously presented to the Museum the large collection which had been on loan from him for a considerable time past in the Society's Museum, and which included cases containing a series of mineral teeth, with the maker's name attached, from 1815 to 1863; also gum-work blocks of moulds for teeth, old-fashioned instruments, &c.

On the motion of the President, the meeting recorded its sense of gratitude to Mr. Hepburn for his handsome donation, especially as many of the objects included were unique, and there were many others, duplicates of which were only to be met with in similiar Museums.

The following members were elected office-bearers for the forthcoming session, 1887-8:—*President*—W. H. Williamson, M. B., C. M., L. D. S. Edin., D. D. S. *Vice-Presidents*—Malcolm Macgregor, L. D. S. Edin.; J. Moore Lipscomb, L. D. S. Eng. *Hon. Treasurer*—G. W. Watson, L. D. S. Edin. *Curator and Librarian*—J. Stewart Durward, L. D. S. Edin. *Hon. Sec.*—John S. Amoore, L. D. S. Eng. *Councillors*—James Mackintosh; W. Bowman Macleod, L. D. S. Edin.; Walter Campbell, L. D. S. Eng.; Rees Price, L. D. S. Eng.

Mr. Andrew Wilson, L. D. S. Edin., read a paper, entitled—

### THE PREMOLARS IN MAN.

The premolars in man are normally two pair in each jaw, and as they have two cusps, more or less decided, they are more frequently called bicuspids.

The latter name is, however, far from definite, as we occasionally, although rarely, have bicuspid central and lateral incisors, and also bicuspid canines, the extra cusp in these being due to an enormous development of the tubercle or prominence in the centre of the cingulum. The forms of the premolars in the upper jaw differ very considerably from those in the lower, and while in the latter the distinction between the first and second is decided, there is little comparatively between the two upper. There being, so far as I am aware, no description of the upper premolars in any of the dental or anatomical works in our language * sufficiently accurate as to enable one to distinguish even the teeth of the two sides, I will, with your permission, begin by endeavoring to give one, taking the first premolars as that best marked. As in all teeth having a grinding surface, we have in the human premolars five surfaces, namely: labial, lingual, mesial, distal, and grinding.

The labial surface is broadest between the mesial and distal angles of its grinding margin; it tapers somewhat abruptly towards the neck, and is convex longitudinally and still more so transversely. The length from the neck to the mesial angle is much less than that to the distal, and it tapers very abruptly from these angles to the apex of the labial cusp, which is placed decidedly to the *distal* side of the tooth, the slope from the apex to the mesial angle being thus much longer and greater than that to the distal angle.

A well-marked ridge passes upwards from the apex and merges in the general surface about half-way up; so leaving two lateral depressions, that toward the mesial angle being the larger and more pronounced; occasionally it is deeply grooved.

The lingual surface may be said to be composed wholly of the cusp and its base. It is shorter than the labial, but is much more convex, both longitudinally and transversely. It terminates in the lingual cusp, the apex of which lies *close to the mesial* side of the tooth.

The mesial surface is at its grinding margin nearly flat, but a little from that it becomes *concave*, the depression being greatest toward the labial margin and neck.

The distal surface is much larger than the mesial; is convex transversely and still more so longitudinally.

The grinding surface is irregularly quadrate, its labial

---

* A small work in German, "Anatomie des menschlichen Gebesses," by E. Muhlreiter, Leipzig, 1870, gives a very detailed and, judging by the engravings, extremely accurate description of the human teeth.

and distal sides being greater than its lingual and mesial respectively. Its centre is occupied by a deep transverse depression, somewhat cresentic in form, the concavity being towards the labial side. From the apex of the labial cusp a well-marked ridge passes upward, ending abruptly in the depression, and occasionally a much fainter one passes from the lingual cusp.

The mesial and distal sides are bounded by a rounded ridge or parapet, continuous with the lingual cusp, that on the mesial side being usually broken by the transverse depression.

The peculiarly irregular form of the grinding surface is best seen if a pair be placed with their mesial sides in contact.

There may be either one, two (labial and lingual), or three (two labial, one lingual, as in the Simiadæ) roots, two being the most frequent, three being comparatively rare. The divisions may occur at any part, and the divergence of the roots may be very decided.

When the root is single, both its mesial and distal surfaces are longitudinally grooved, that on the former being the deeper, and there is not unfrequently a slightly marked one on the labial surface.

The pulp cavity in the crown follows the contour of the tooth, having corona corresponding to the cusps. At the neck the canal is much compressed, especially in the middle, becoming so much so in the grooved root of single rooted teeth as to be practically two canals united by a mere fissure.

The description of the second differs in a few points. In it the labial and lingual surfaces are nearly equal, the former is less V-shaped, the depressions on each side of the labial ridge are almost obsolete, and the root is more frequently single.

Abnormal forms in these teeth seem rare. I have met with one case in which the first on both sides simulated the first lower, in that the lingual cusp was rudimentary, and was connected with the labial by a well-marked ridge, on each side of which was a slight depression. We have also cases in which the first is a geminated tooth (union with a conoid tooth on its mesial side), three of which I show. I have also met with one case in which a second has assumed the conoid form. *

The second is also very frequently smaller than the first, the difference in some cases being very marked. Both seem to be more liable than other teeth to become more or less rotated during eruption, the second being not unfrequently semi-rotated.

Of the lower premolars we have already very good descriptions; in them, as in all the other teeth (the upper premolars alone excepted), the most prominent point of the convexity of the labial surface is towards the mesial side, to which also the labial cusp points, and their lingual surface is much less convex than the labial, just the reverse of the uppers (this also applies to the molars).

They have almost always single roots, but we occasionally meet with some having two, which are labial and lingual, as in the upper (in the Simiadæ, where two is the normal number, they are mesial and distal, as in the molars). Abnormal forms seem rarer than even in the upper. I have met with the first, a geminated tooth (two firsts), and also cases in which firsts and seconds were very much flattened, the mesio-distal diameter being much the largest.

A form of the second, in which the lingual cusp is divided by a notch into two sub-equal cusps, is extremely common.

The extreme convexity longitudinally of the labial surface in the first (the cusp being almost over the centre of the tooth) is much more strongly marked in the anthropoid apes, and I think can be traced to the very peculiar form seen in the macacques and baboons, in which a large surface is opposed to the formidable upper canine.

* Since writing this paper, I have met with another abnormal form. In it there are on the labial surface, besides the ordinary cusp, two others, one on each side, springing from about the middle of the surface.

The premolars present in man are usually given as representing the third and fourth of the typical placental mammalian dentition, but I am much more inclined to regard them as the second and third, for the following reasons:—

In those mammals in which we have the typical number, the first is usually more or less rudimentary in form, and almost, if not quite, invariably has had no deciduous predecessor. It is this tooth which I consider represented in man by the conoid portion of a geminated first.

In the few cases on record of a normally formed supernumerary premolar in the human dental arch, it is to the distal side of the normal second, and in the only case I have met with (lower jaw) that on the one side erupted after the extraction of the first molar, while that on the other side only did so after the removal of both the first and second molars. I am inclined to consider these rudimentary, and almost conoid teeth, which we not unfrequently meet with in the upper jaw, to the buccal side of the normal series. As supernumerary premolars (the fourth typical) we have them between the second premolar and second molar, between the first and second, and even between the second and third. They also occur as conoid teeth geminated with the first molar (on their buccal surface.)

The tendency to dwarfing in the second, and its being occasionally conoid, also point to the same conclusion.

In the platyrrhine monkeys, where three premolars are normally present, the first differs in form from the other two, which are alike, and as they have three molars in their temporary dentition, I regard their successors, the premolars, as the second, third and fourth of the typical dentition.

I may notice, in passing, that the teeth generally in these apes are much more human in form than those in the old world monkeys, the anthropoids excepted.

Those members who take a special interest in this subject will have remarked that I have made no reference to the extraordinary case recorded (with illustration) in the Association Journal for March, 1886, in which there are six premolars, all well formed, on the one side, the normal number being on the other. My reason for doing so is that there are so many points in which it is abnormal, and so little is known of its history, that I could not venture to draw any deductions from it.

As regards the number, it is just double of any other case I know of on record. Even when we include the lower mammals, very few cases are recorded of five premolars on one side. Owen figures one in an Indian boar,* another is figured by De Blainevjlle in one of the long-muzzled canidæ, † and I exhibited another in an Australian dingo to this Society last session; but these are all in species in which four is the normal number, not two, as in man, and the supernumerary is only a duplicature of the first, the most rudimentary of the series, and having a temporary predecessor. In this case none are rudimentary.

As I have already remarked, unfortunately little is known of the history of the case; nothing in reference either to the number and position of the temporary molars or order of eruption of the premolars and first molar.

Supposing there was no excess of temporary molars and that their arrangement was normal, the germs of the two extra premolars, which are *in* the arch, must have been developed and in possession of the space belonging to the first permanent molar at a very early period—a state of matters which would most likely have involved their eruption in advance of the molar. Again, as there was no abnormality in the lower teeth, one would have liked to learn how these articulated with the upper.

The case is stated to have been complicated by cleft palate, but judging from the illustration; one would hardly have thought so.

---

* Odontography, Plate 141, Fig. 3.

† C. Tomes' Manual, 2nd ed., p. 386.

In conclusion, I will only add that I sincerely trust the time is now past when such a case, or any approaching it, will be allowed by any dental surgeon to lie unrecorded for years, as this one was.

DISCUSSION.

Dr. Smith said that, not previously knowing the drift of Dr. Wilson's paper, he felt some difficulty in complying with the Chairman's request to open the discussion upon this abstruse and interesting subject. It was something new in the literature of dentistry, as in no work he knew of in the English language was there any such admirable description given of the anatomical peculiarities and distinctive characters of the premolars in man. A dissertation upon these teeth was beset with many difficulties. It would be recollected that only one of the four classes of teeth met with in the mammalia was really scientifically defined, and that was the incisors. Shape, number, size, and other matters, which were liable to vary and become grounds of dispute, were not maintained as the main definitions distinguishing the incisors, these teeth being always held to be simply the teeth contained in the intermaxillary bones. In man the bicuspids were described as the surviving representatives of the premolar teeth figuring in the archetypal formula of the mammals. But, even in such formula, what precise characters constituted or were essential to a premolar tooth was a difficult matter to determine. They were not in every case the successors of the milk molars, as they were in man. They varied in size, and shape, and number, as well as in having predecessors, or having successors, and even in their presence, to a considerable extent, throughout the animal kingdom. Even the canine tooth itself—the first or anterior of those in the true maxilla—might sometimes be fairly classed among the premolars, as in those cases where all its typical characters were wanting, and were found in some other class of tooth, such as the incisor, and which occurred in various well known instances among the lower animals. These remarks had suggested themselves on hearing Mr. Wilson's interesting allusions to the variety in certain points of formation and number among these teeth when occurring in various animals, as relating to certain abnormalities in the number of these particular organs, and the multiplication of their fangs occasionally met with in the human subject. Wherever the full number or perfect development of these or other systematic parts throughout any class of animals departed from what appeared to be an established typical formula, it might, according to modern research, be assumed as in all probability due to the influence of what was known as diminished functional activity, or to what was called adaptive modification, leading to survival of the fittest—a natural law exemplified in the case of individual animals where certain structures, whose function had ceased, disappeared; such as in the ductus arteriosus, or thymus gland, after birth; in the atrophy of muscle from disuse; or of the optic nerve, where certain causes have led to blindness. These were all interesting and instructive points suggested by Mr. Wilson's valuable and exhaustive communication. The plaster models, also, which he had exhibited as illustrating his views, were excellent examples of that structural conformation in special and differentiated teeth, such as those of the heterodonts, which had led to the hypothesis that they were derived merely from some fusion of the simpler forms of these organs, as met with in the homodonts. Altogether the paper was one which merited the warmest thanks of the Society.

Mr. Watson described a lower right wisdom tooth of abnormal shape, which had been extracted at the hospital, with considerable difficulty, by one of the students. On the coronal portion of the inner margin of the posterior lingual root arose a somewhat longish oval protuberance, projecting backwards and slightly outwards—constricted at its attachment, but swelling out towards its extremity. It was covered by a thick and highly vascular membrane (enamel membrane), the whole being beneath the surface of the gum when the tooth was in position in the mouth. At the base of the protuberance, and somewhat between the normal roots, was situated a third small and somewhat flattened root. He had made a section through the abnormal portion, and used part of it for a micro preparation, from which was taken a photomicrograph. On examination after section, the pulp chamber was found to have passed some distance into the protuberance, which, in fact, was analagous to the crown of the tooth. Of the general appearance of the tooth, the members would be able to judge from the remains of the tooth, and also from a model of the tooth in its entirety—and the nature of its histological structure could be better understood by reference to the photo-micrograph. The probable physiological explanation of this malformation was that the germ of a supernumerary tooth had been developed in close approximation to the third molar, with which it eventually coalesced, the small extra root properly belonging to the supernumerary, and the two teeth possessing pulp chambers in common.

Mr. Watson also called attention to the suitability of photomicrographs for teaching purposes, owing to the great improvements in objectives within the last few years, with which most beautiful and satisfactory results could now be obtained. In one of the specimens exhibited—a full-length section of a lower first bicuspid, with an odontome upon the root—it was impossible a few years ago to get a lens to take in more than the crown of the tooth, but now it could be represented in its entirety.

Photo-micrographs of osteo-dentine, dentine of repair, &c., were also shown.

Mr. Macleod made a short communication on the subject of Cunningham's method of facing hard rubber plates with metal. He said the process has for its object the placing between the palatine surface of vulcanite dentures and the mucous membrane of the mouth a layer of gold, which, being a good conductor of heat, will reduce the tendency to inflammatory action which vulcanite is said to induce. Several attempts have previously been made to line the surface of plates by means of gold leaf, &c., but these have all proved failures, owing to the readiness with which the gold lining scaled from the plate, to say nothing of the difficulty of carving the leaf in a uniform and unbroken surface round isolated teeth and up to the margin of the finished plate. Cunningham's process has none of these defects. Whether it will reduce the liability to congestion or not, it presents an attractive and workmanlike finish, and a surface which is more readily kept clean, and on these two points deserves our present commendation, leaving the more debatable one of avoidance of inflammation to be determined by experience.

Mr. Macleód then illustrated the process on prepared models, and the finished work by specimens made by Mr. Cunningham and himself.

Mr. Wilson said that he could not see how any benefit could be derived from the heat-conducting property of the gold lining, as it was covered on its lingual surface by a thick layer of vulcanite, its only free surface being practically the edge exposed at the palatal margin. The metallic layer might possibly assist in the case being kept clean, but the whole affair had an unpleasant odour of humbug about it. Having had personal experience of a vulcanite surface, both coloured and uncoloured, in contact with the palate for fully five-and-twenty years, he had found no uneasiness which could be traced to its being a non-conductor of heat.

Dr. Smith said he was not quite sure how the conducting power of a metal would be influenced by being coated on one side by vulcanite in a cavity such as the mouth, where the temperature was not always uniform. The metal might act as a conductor of heat in two ways— it might convey heat from the palate, but on taking hot food into the mouth it would act in the opposite manner. This the vulcanite coating might obviate. As for irritation being caused by vulcanite, it no doubt might occur in exceptional cases, but he had seen very similar irritation. occasioned by gold plates, and long ago by the old fashioned bone sets—in certain patients.

Mr. Brownlie said there can be no question about the improved appearance which Mr. Cunningham's process gives to vulcanite work—converting what is, in its best fitting form, an unpleasant-looking surface into a "thing of beauty." In his humble opinion its merit ends there, however.

From the notices which have been given of this process, and the statements made in connection with it from time to time, it appears to have been devised to cure, and has been thought to cure, a condition with which every practitioner is more or less familiar, but which is not due to wearing vulcanite. He could recall as many cases of the like condition when gold was worn as when vulcanite was the base. Want of cleanliness explains much of it, but not all. In some cases they must look upon it as a condition peculiar to the individual, aggravated by continuous wear and a close fit.—*London Dental Record.*

---

